# Complex landscape of somatic copy number alterations in head and neck paragangliomas

**DOI:** 10.3389/fendo.2026.1841009

**Published:** 2026-08-14

**Authors:** Vladislav S. Pavlov, Maria S. Fedorova, Turpal-Ali S.M. Elnukaev, Dmitry V. Kalinin, Elena A. Pudova, Irina V. Katunina, Zulfiya G. Guvatova, Anastasia A. Kobelyatskaya, Andrey D. Kaprin, Anna V. Kudryavtseva, Anastasiya V. Snezhkina

**Affiliations:** 1 Engelhardt Institute of Molecular Biology, Russian Academy of Sciences, Moscow, Russia; 2 Vishnevsky Institute of Surgery, Ministry of Health of the Russian Federation, Moscow, Russia; 3 National Medical Research Radiological Centre of the Ministry of Health of the Russian Federation, Obninsk, Russia; 4 Scientific and Educational Resource Center for Innovative Technologies of Immunophenotyping, Digital Spatial Profiling and Ultrastructural Analysis, Patrice Lumumba Peoples’ Friendship University of Russia (RUDN University), Moscow, Russia

**Keywords:** head and neck paragangliomas, copy number alterations, allele-specific analysis, tumor suppressor genes, oncogenes, genomic instability

## Abstract

**Background:**

Copy number alterations (CNAs) drive cancer by amplifying oncogenes and deleting tumor suppressor genes. Although CNA patterns are well-studied in common cancers, they remain poorly characterized in rare tumors.

**Methods:**

In this study, we employed an allele-specific copy number analysis using ASCAT on 25 head and neck paragangliomas (HNPGLs) with high tumor cell purity (≥70%) and available clinicopathologic and mutation data.

**Results:**

The majority of HNPGLs exhibited a near-diploid state. The recurrent somatic CNAs were predominantly hemizygous deletions, frequently affecting chromosomal regions 7q11, 1p36, 1p34, and 1p21.1-1p13.2, and involving key tumor suppressor genes associated with paragangliomas/pheochromocytomas (PPGLs), including *SDHB*, *SDHD*, *ATRX*, *SDHAF2*, *FH*, *MEN1*, *SDHA*, and *VHL*. Frequent homozygous and hemizygous deletions were also observed in the tumor suppressor genes *BCL10*, *PHF6*, *RPL5*, and *STAG2*. Whole-gene amplifications were identified in several PPGL-associated oncogenes, such as *BRAF*, *IDH1*, *PDGFRA*, and *KIT*. Additionally, we identified concurrent mutations and allelic imbalances, suggestive of potential wild type allele loss in several novel tumor suppressor genes (*CARS* and *PRDM2*), which may point to a role in tumor pathogenesis.

**Conclusion:**

Collectively, our study reveals a complex landscape of somatic CNAs in HNPGLs. The presence of recurrent alterations in hotspot genomic loci and PPGLs-associated genes suggests that genomic instability may be a significant contributing factor to tumor development and progression.

## Introduction

Copy number alterations (CNAs) are defined as the large-scale chromosomal alterations, including deletions, duplications, and further multiplications, which are categorized as structural variations within the framework of mutation classification (1). The size of CNAs ranges from 50 base pairs (less than an average exon size) to several million base pairs of DNA. CNAs can be copy-neutral or deleterious, which have the potential to result in gene dosage alterations, gene or regulatory region interruptions, fusion transcripts, position and synergistic effects (2, 3). CNAs are prevalent in cancer and play a pivotal role in tumor initiation and progression (4). It has been established that an average of 30% of the human cancer genome is characterized by somatic amplification and deletion of DNA segments with a mean length of approximately 4 Mb (5, 6). Different cancer types can manifest a distinct genomic landscape of somatic CNAs that can impact tumor pathogenesis, patient prognosis, and response to anticancer treatment (7). However, despite the well-established role of CNAs in tumorigenesis, their biological significance in the development of rare cancers remains largely unexplored.

Head and neck paraganglioma (HNPGL) is a rare human neoplasm that belongs to a group of neuroendocrine tumors collectively called paragangliomas and pheochromocytomas (PPGLs) (8). The genetics of PPGLs have been the focus of extensive study over the past few decades. This has led to the identification of a list of tumor susceptibility genes (*SDHx*, *NF1*, *FH*, *VHL*, *RET*, *MAX*, and others) and associated molecular pathways (9). Hereditary predisposition was thought to play a significant (up to 40%) role in the formation of PPGLs (8). Germline mutations in *SDHx* genes have been identified as the most prevalent genetic cause of hereditary HNPGLs (10). However, the molecular genetic bases of apparently sporadic PPGLs remain largely elusive, and even in hereditary cases, the spectrum of somatic CNAs and their functional contributions to tumor development are still incompletely understood.

Several studies have begun to address CNAs in HNPGLs. Sevilla et al. used microarray CGH to identify recurrent deletions at 1p, 1q, and 11q in both familial and sporadic tumors (11), whereas Cama et al. performed high-density genome-wide copy number variation analysis on 24 cases, identifying 104 genes significantly targeted by tumor-associated alterations and revealing dysregulation of the NOTCH signaling pathway (12). Building on these findings, the present study provides a comprehensive profile of somatic CNAs in HNPGLs using allele-specific copy number analysis (ASCAT) applied to 25 high-purity tumor samples. This approach allowed us to characterize ploidy changes, distinguish between hemizygous and homozygous deletions, and identify recurrent losses of tumor suppressor genes as well as amplifications of clinically relevant oncogenes. Our findings expand the current understanding of HNPGL pathogenesis and establish preliminary data to support future investigations into CNA-based biomarkers for diagnosis, prognosis, and targeted therapy.

## Materials and methods

### Patients and specimens

The study incorporated 25 tumors and their corresponding normal tissues. All samples were derived from patients diagnosed with HNPGLs. Formalin-fixed paraffin-embedded (FFPE) tumor samples, along with paired normal controls (lymph node or whole blood), were collected at the Department of Pathology. The study was conducted in accordance with the Declaration of Helsinki (1964) and was approved by the Ethics Committee. Written informed consent was obtained from all individual participants included in the study.

Clinical and demographic characteristics are reported at the patient level (n=21). Genomic analyses were performed at the tumor level (n=25), recognizing that tumors from the same patient are not fully independent. When reporting frequencies of genomic events, we refer to the proportion of tumors unless otherwise specified. Tumors were classified as apparently sporadic if no pathogenic mutations were identified in PPGL susceptibility genes, such as *SDHx, SDHAF2, VHL, RET, FH, NF1, IDH1/2, TMEM127, MAX*, *MDH2, SLC25A11, EGLN1/2, MEN1, EPAS1, GOT2, DLST*, and *SUCLG2*. Tumors with mutations in any of these genes were designated as *SDHx*-mutated or genetically defined tumors.

### DNA isolation

DNA from FFPE tissues was manually isolated using the High Pure FFPET DNA Isolation Kit (Roche, Switzerland). Blood DNA was automatically extracted using the MagNA Pure Compact Nucleic Acid Isolation Kit on a MagNA Pure Compact Instrument (both from Roche, Switzerland). DNA quantity and quality were assessed using a Qubit 4.0 fluorometer (Thermo Fisher Scientific, USA) and an Agilent 2100 Bioanalyzer (Agilent Technologies, USA), respectively. Subsequently, 1 µg of DNA was used for exome library preparation and sequencing.

### Whole exome sequencing

Exome libraries were prepared in accordance with the manufacturer’s instructions using the TruSeq Exome Library Prep Kit (Illumina, USA). Library preparation consisted of the following key steps: mechanical genomic DNA fragmentation using a Covaris S 220 (Covaris, USA), end-repair and fragment length optimization, adenylation of 3’ ends, adapter ligation, DNA fragment enrichment, and library validation. Subsequently, pooled libraries with distinct indexes were hybridized to capture probes to enrich for protein-coding regions. The target-enriched DNA fragments were then purified and subjected to limited-cycle amplification. Library quantity and quality were assessed using the Qubit dsDNA HS Assay Kit on a Qubit 4.0 fluorometer and the Agilent High Sensitivity DNA Kit on an Agilent 2100 Bioanalyzer, respectively. The sequencing of exome libraries was performed on a NextSeq 500 System (Illumina, USA). Paired-end sequencing was conducted with a read length of 76 base pairs in each direction.

### Allele-specific copy number analysis

The evaluation of sequencing quality metrics was performed using the FastQC software (v. 0.11.9) (13). Adapter sequences, low-quality bases and reads with a quality score (Q) of less than 30 were removed using Trimmomatic (v. 0.39) (14). The obtained reads were then aligned on the human reference genome assembly GRCh37/hg19 using the BWA-MEM algorithm (v. 0.7.17) (15). The resulting SAM files were processed to create BAM files. The GATK MarkDuplicates (v. 4.2.4) tool was utilized to mark duplicate reads in sorted BAM files (15).

For copy number analysis, Log R Ratio and B Allele Frequency files were generated from curated BAM files. The proportion of normal cells within each tumor sample was estimated, and only samples containing less than 30% normal cells (i.e., >70% tumor cells) were retained for further analysis. These were subsequently imported into the ASCAT (v. 3.2.0) algorithm (16). ASCAT was utilized to infer both the tumor ploidy and the fraction of tumor cells. To mitigate technical noise inherent to the ASCAT algorithm and to reduce false-positive calls resulting from allelic imbalance artifacts, we applied a fragment-length filter based on median absolute deviation of LogR values. Only somatic copy-number alterations with a genomic length of at least 5 megabases (Mb) were retained for downstream analysis. This threshold was empirically determined based on the noise profile of our dataset. The genomic coordinates of all identified CNAs were annotated to map them to specific chromosomal regions. The Ensembl database (release corresponding to GRCh37) was utilized to assign each alteration to its respective chromosome cytoband location and genes that overlap the region. In order to prioritize biologically and clinically significant findings, the list of genes located within CNA regions was cross-referenced against the Cancer Gene Census (17) from the COSMIC database. Allele-specific CNAs (A:B) were categorized according to the baseline ploidy and chromosomal ratio as follows (**Table 1**). In a near-diploid genome (1:1 chromosomal ratio), CNA types included hemizygous deletion (1:0), homozygous deletion (0:0), amplified loss of heterozygosity (LOH, >1:0), asymmetric amplification (>1:≥1, where A ≠ B), and focal balanced amplification (>1:>1, where A = B). In a near-tetraploid genome with a 2:2 chromosomal ratio (tumor ID: 066tc), the corresponding categories were hemizygous deletion (1:0 or 2:0), homozygous deletion (0:0), amplified LOH (>2:0), asymmetric amplification (>2:≥2, A ≠ B), and focal balanced amplification (>2:>2, A = B). In a near-tetraploid genome with a 3:1 chromosomal ratio (tumor ID: 065tc), the categories were defined as hemizygous deletion (1:0, 2:0, or 3:0), homozygous deletion (0:0), amplified LOH (>3:0), asymmetric amplification (>3: ≥1, A ≠ B), and focal balanced amplification (≥3:≥3, A = B). Mutation analysis and variant annotation of COSMIC genes were carried out as previously described (18).

**Table 1:** Classification of allele-specific CNAs by tumor ploidy.

CNA type	Diploid (1:1 ratio)	Tetraploid (2:2 ratio) [tumor ID: 066tc]	Tetraploid (3:1 ratio) [tumor ID: 065tc]
Hemizygous deletion	1:0	1:0 or 2:0	1:0, 2:0 or 3:0
Homozygous deletion	0:0	0:0	0:0
Amplified LOH	A:0 (A > 1)	A:0 (A > 2)	A:0 (A > 3)
Asymmetric amplification	A:B (A ≠ B A > 1, B ≥ 1)	A:B (A ≠ B A > 2, B ≥ 2)	A:B (A ≠ B A > 3, B ≥ 1)
Focal balanced amplification	A:B (A = B > 1)	A:B (A = B > 2)	A:B (A = B ≥ 3)

### Microsatellite analysis

In order to confirm the loss of heterozygosity at the loci of the *SDHB* [1p36.13] and *SDHD* [11q23.1] genes, the following dinucleotide microsatellite markers were utilized: D1S228 [1p36.21], D1S507 [1p36.21], D1S436 [1p36.21], D1S552 [1p36.13], D1S199 [1p36.13], D11S1339 [11q22.1], D11S927 [11q22.3], D11S5030 [11q23.1] and D11S1347 [11q23.1]. The markers were analyzed in tumor samples and their corresponding paired normal tissues (blood or lymph node). The DNA from the paired samples was diluted to an equal concentration of 50–100 ng. The process of PCR amplification was conducted using primer sets incorporating fluorescently labelled forward primers at the 5’ end and unlabelled reverse primers (Sintol, Russia), and Tersus Plus PCR kit (Eurogen, Russia). Reactions were performed on a T100 thermal cycler (Bio-Rad, USA) in accordance with the following protocol: an initial denaturation at 95 °C for 5 min; 30 cycles of 94 °C for 30 s, 48-55 °C for 30 s, and 72 °C for 30 s; followed by a final extension at 72 °C for 7 min.

The sizes of the PCR products were verified on a 2% agarose gel and subsequently purified using KAPA Pure Beads (Roche, Switzerland). Fragment analysis of the amplicons was performed on a NANOFOR-05 instrument (Sintol, Russia). LOH visualization and analysis were conducted using the GeneMarker software (SoftGenetics, USA).

### Statistical analysis

The Mann-Whitney U test and Kendall’s rank correlation coefficient were used to statistically analyze the relationship between tumor ploidy and сlinicopathologic characteristics of patients. Statistical analysis was performed using STATISTICA 10 (Statsoft, Inc., USA). Fisher’s exact test was used to assess the statistical significance of copy number alterations between *SDHx*-mutated and non-*SDHx*-mutated tumors.

## Results

### Frequent CNAs in HNPGLs

The analysis of allele CNAs was performed using the ASCAT method based on whole-exome sequencing data from paired tumor and normal tissues. After quality filtering, 25 tumors from 21 patients with HNPGLs were included in the analysis (**Table 2**). Tumor ploidy varied from 1.58n to 4.54n and exhibited no significant associations with mutations in the PPGLs-associated genes (**Supplementary File 1**). Due to the limited number of cases with metastasis, recurrence, or multifocality in the studied cohort, correlation with ploidy could not be reliably evaluated.

**Table 2:** Clinicopathologic characteristics of studied patient with HNPGLs.

Characteristics	Value
Age (years)	
Median	55
Range	19-67
Sex	
Male	19% (4)
Female	81% (17)
Tumor clinical feature	
Multifocal	14% (3)
Recurrent	9.5% (2)
Metastasis	9.5% (2)
Tumor localization	
Carotid artery bifurcation	68% (17)
Along the vagal nerve	32% (8)
Mutation in susceptibility gene	
*SDHx*	62% (13)
No mutation	38% (8)

**Figure 1:**
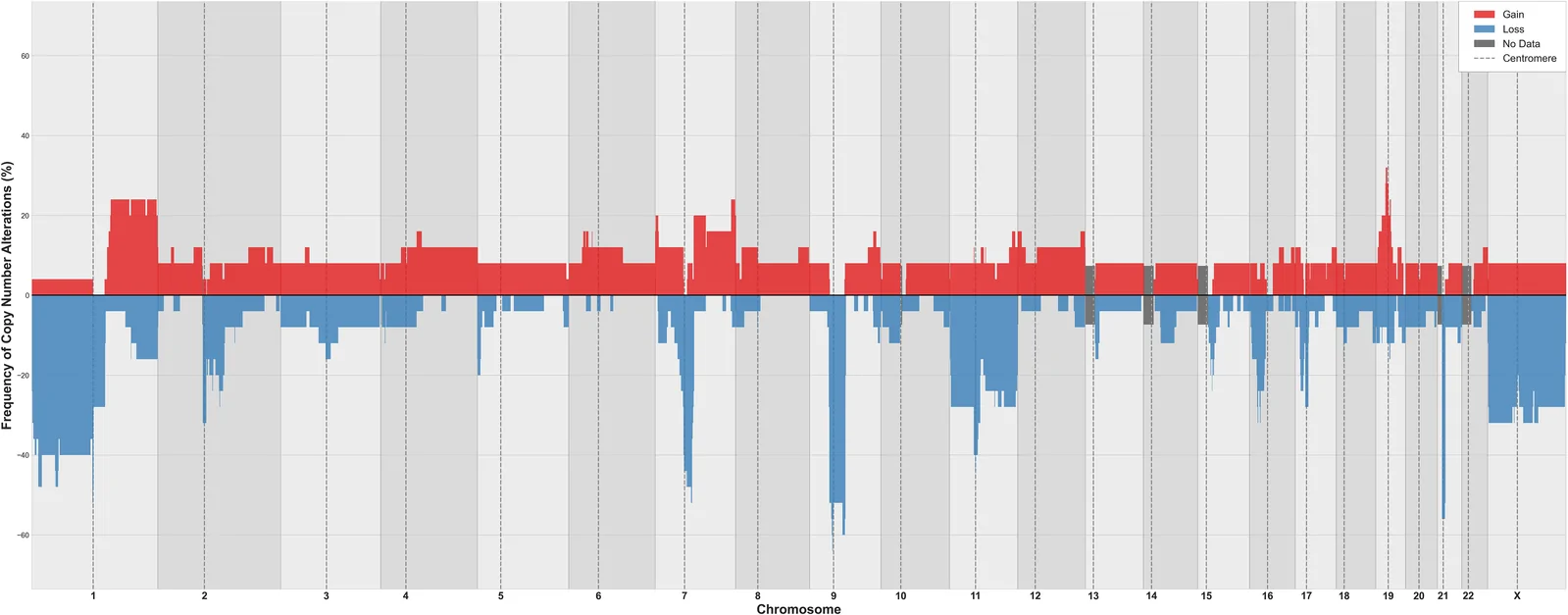
Frequency and distribution of copy number alterations in HNPGLs. The plot displays genome-wide distribution of copy number alterations ≥5 Mb and their corresponding frequencies across the tumor samples analyzed. Blue indicates deletions (loss), whereas red indicates amplifications (gain).

Approximately half of the tumors exhibited recurrent somatic CNAs, with deletions being the predominant type. These deletions ranged in size from 5 Mb to 144 Mb and were observed in 8–48% of tumors (**Figure 1**; **Supplementary File 2**). Hemizygous deletions involving cytobands 7q11, 1p36, 1p34, and 1p21.1–1p13.2 were present in approximately 30-50% of HNPGLs. Homozygous deletions were predominantly located in regions 9p11.2–9q21.11 and 21p11.1–21q11.2. Across all ASCAT−derived segments meeting our ≥ 5 Mb threshold, we detected no amplified LOH events, nor any cases of copy number−neutral LOH.

Amplifications ranged in size from 5 Mb to 71 Mb and were observed in approximately 15% of the HNPGLs (**Figure 1**; **Supplementary File 2**). The most common asymmetric allele amplifications were identified within cytobands 9q33.3–9q34.11, 7q36, and 1q41–1q42.11. Focal balanced amplifications were observed only in a few samples, predominantly in the near-tetraploid tumors 065tc and 066tc (**Supplementary File 1**).

Compared with *SDHx*−mutated tumors, apparently sporadic (non−*SDHx*−mutated) HNPGLs exhibited recurrent asymmetric allele amplifications within regions 1q31–1q44, 11q23–11q25, and 3p21–3p14 (ranging from 7.5 to 25 Mb; p ≤ 0.01; **Supplementary Table 1**; **Supplementary File 3**). Notably, a homozygous deletion at 1p32.3–1p21.1 was also significantly associated with the non−*SDHx*−mutated phenotype (p = 0.02). However, these findings require additional validation in an expanded sample set, as the Benjamini–Hochberg-adjusted *q*-value for the identified loci did not reach the threshold for statistical significance (*q* = 0.3).

### Oncogene and tumor suppressor gene CNAs

CNAs affecting at least one oncogene and/or tumor suppressor gene were identified in 24 out of 25 tumors analyzed (**Supplementary Figures 1**, **S2**; **Supplementary File 3**). Frequent hemizygous deletions affecting the tumor suppressor genes *SPEN*, *CASP9*, *PRDM2*, *SBDS*, *ARHGEF10L*, *SDHB*, *ARID1A*, *CDKN2C*, and *ID3* were observed in approximately half of the tumors (**Supplementary Figure 1**; **Supplementary File 3**). Analysis of the established PPGL gene panel (19) revealed hemizygous deletions in eight genes. The most frequently altered were *SDHB* (44%, 11/25), *SDHD* (28%, 7/25), and *ATRX* (28%, 7/25), followed by *SDHAF2* (20%, 5/25), *FH* (16%, 4/25), *MEN1* (12%, 3/25), *SDHA* (8%, 2/25), and *VHL* (8%, 2/25) (**Tables 3**, **4**). In tumors exhibiting germline *SDHB* or *SDHD* mutations, microsatellite analysis revealed loss of heterozygosity near the gene locus, thereby confirming biallelic gene inactivation via somatic deletion of the wild-type allele (**Supplementary Figure 3**; **Supplementary File 3**). Co-occuring missense mutation and hemizygous deletion were observed in several additional tumor suppressor genes, including *CARS*, *CBLB*, *PRDM2*, and *SMARCA4*, suggesting potential biallelic gene disruption (**Table 4**). Homozygous deletions were detected in a small subset of tumors (10-12%) and affected the tumor suppressor genes *ATP2B3, BCL10, PHF6, RPL10, RPL5*, and *STAG2*.

**Table 3:** Copy number alterations in PPGLs-related tumor suppressor genes and oncogenes identified in HNPGLs. .

Tumor ID	Location	Feature	Ploidy	*SDHx* mutation	Mutation status	Other PPGLs-related gene mutation	Hemizygous deletion of PPGLs-related TSGs	Homozygous deletion of TSGs	Amplification of PPGLs-related oncogenes
001tc1	CPGL	Mult	2.01	*SDHD*	Germ	No	-	-	-
001tc2	CPGL	Mult	1.99	*SDHD*	Germ	*TP53*	-	-	-
001tv	VPGL	Mult	2.18	*SDHD*	Germ	No	*SDHD*	-	-
024tc	CPGL	-	1.98	No	-	No	*SDHB*, *ATRX*	*BCL10, RPL5*	-
026tv	CPGL	-	1.97	No	-	No	-	-	-
062tc	CPGL	-	1.97	*SDHD*	Germ	No	-	-	-
065tc	CPGL	-	4.29	No	-	No	-	-	*KIT, PDGFRA*
066tc	CPGL	-	4.54	No	-	No	*SDHB*	-	*IDH1, KIT, PDGFRA*
067tc	CPGL	-	1.81	*SDHD*	Germ	No	*SDHB*, *SDHD*, *SDHAF2*, *MEN1*	-	-
072tc	CPGL	-	1.80	No	-	*PCK1*	*SDHB*	*ATP2B3, BCL10, PHF6, RPL10, RPL5, STAG2*	-
082tc1	CPGL	Mult	2.21	*SDHD*	Germ	No	*ATRX*	*PHF6, STAG2*	-
082tv	VPGL	Mult	2.06	*SDHD*	Germ	No	*ATRX*	-	*IDH1*
100tc	CPGL	-	1.73	*SDHD*	Germ	No	*SDHB*, *SDHD*, *SDHAF2*, *ATRX*, *MEN1*	-	*BRAF*
101tv1	VPGL	Rec	1.93	*SDHB*	Germ	*TERT*, *OGDHL*	*SDHB*, *FH*	-	-
101tv2	VPGL	Rec	1.95	*SDHB*	Germ	*TERT*	*FH*	*ATP2B3*	-
103tc	CPGL	-	1.93	*SDHD*	NA	*TP53*	*SDHB*, *SDHA*, *ATRX*	*RPL10*	-
105tc	CPGL	-	1.77	No	-	No	*SDHB*, *VHL*	*BCL10, RPL5*	*BRAF*
106tc	CPGL	Met, rec	1.97	*SDHB*	Germ	*PC*, *TERT*	*SDHB*	-	-
118tv	VPGL	-	2.06	No	-	*L2HGDH*	-	-	-
120tv	VPGL	-	1.58	*SDHD*	Germ	No	*SDHD*, *SDHA*, *SDHAF2*, *ATRX*	-	-
123tv	VPGL	-	1.61	No	-	No	*SDHB*, *SDHD*, *FH*	-	-
125tv	VPGL	-	2.67	*SDHB*	Germ	No	*SDHB*, *SDHD*, *SDHAF2*, *FH*, *MEN1*	-	-
143tc	CPGL	-	2.15	*SDHD*	Germ	*LRFN4*	*SDHD*, *SDHAF2*, *VHL*	-	-
149tc	CPGL	Met	2.01	*SDHD*	Som	*KMT2D*	-	-	-
157tc	CPGL	Mult	2.28	*SDHD*	Germ	No	*ATRX*	-	-

CPGL, carotid paraganglioma; VPGL, vagal paraganglioma; PPGLs, paragangliomas and pheochromocytomas; TSGs, tumor suppressor genes; Mult, multifocal; Met, metastasis; Rec, recurrent; Germ, germline; Som, somatic; NA, data unavailable.

**Table 4:** Tumor suppressor genes and oncogenes affected by both genetic mutations and copy number alterations.

Gene	COSMIC gene	Mutation	Mutation type	Mutation status	VAF	ACMG/AMP 2015 classification	CNA type	Tumor ID
*CARS*	TSG	NM_001751: c.G942T, p.K314N (chr11:3041525, rs150783844)	Mis	Germ	0.79	VUS	Hemi_del	067tc
*CBLB*	TSG	NM_001321788: c.C2702G, p.A901G (chr3:105378061, rs55821768)	Mis	Germ	0.39	VUS	Hemi_del	024tc
*PRDM2*	TSG	NM_012231: c.A2687G, p.Y896C (chr1:14106977, rs756985448)	Mis	Som	0.75	VUS	Hemi_del	105tc
*SDHB*	TSG	NM_003000: c.287-2A>G (chr1:17355233, rs1064794270)	Mis	Germ	0.81, 0.60	P	Hemi_del	106tc, 125tv
*SDHB*	TSG	NM_003000: c.C136T, p.R46X (chr1:17371320, rs74315370)	Nons	Germ	0.46	P	Hemi_del	101tv1
*SDHD*	TSG	NM_003002: c.A305G, p.H102R (chr11:111959726, rs104894302)	Mis	Germ	0.58, 0.65, 0.62, 0.69, 0.53	LP	Hemi_del	001tv, 067tc, 100tc, 120tv, 143tc
*SMARCA4*	TSG	NM_001128844: c.G115A, p.A39T (chr19:11094942, rs764312409)	Mis	Germ	0.45	VUS	Hemi_del	100tc
*CDKN1A*	Oncogene	NM_000389: c.G350A, p.C117Y (chr6: 36652228, rs148679597)	Mis	Germ	0.3	VUS	Asym_ampl	062tc
*HGF*	Oncogene	NM_001010932: c.T667G, p.S223A (chr7:81374380, rs139457161)	Mis	Germ	0.52	VUS	Asym_ampl	123tv

TSG, tumor suppressor gene; Mis, missense; Nons, nonsense; CNA, copy number alteration; VAF, variant allele frequency; VUS, variant of uncertain significance; LP, likely pathogenic; P, pathogenic; Asym_ampl, asymmetric amplification; Hemi_del, hemizygous deletion; Germ, germline; Som, somatic; ACMG/AMP, American College of Medical Genetics and Genomics and the Association for Molecular Pathology.

Whole oncogene amplifications were observed less frequently than deletions of tumor suppressor genes. The most prevalent were asymmetric amplifications involving the oncogenes *AKT3*, *CALR*, *LYL1*, and *PRKACA*, which were present in 16% of HNPGLs (**Supplementary Figure 2**; **Supplementary File 3**). Of the genes in the PPGL panel, amplifications of oncogenes *BRAF*, *IDH1*, *KIT*, and *PDGFRA* were detected in 8% of tumors (**Table 3**). Among all amplified oncogenes identified, *CDKN1A* and *HGF* were additionally characterized by missense mutations within the same tumor samples (**Table 3**).

A detailed analysis of *SDHx*−mutated tumors (n=17) revealed that the tumor suppressor genes *SDHB*, *SDHD* and *ATRX* harbored hemizygous deletions in approximately half of cases (**Table 3**). Deletions affecting *SDHAF2* were observed in 29% (5/17) of these tumors. Notably, the group of apparently sporadic HNPGLs (n = 8) also exhibited frequent hemizygous deletions in *SDHB* (62.5%, 5/8) and *ATRX* (25%, 2/8) genes (**Table 3**).

## Discussion

We examined tumor ploidy in HNPGL samples characterized by the highest purity of tumor cell populations. Approximately half of the HNPGLs exhibited a diploid or near-diploid pattern (1.9–2.15n). A quarter of tumors exhibited hyperploidy (2.18–4.54n) or hypoploidy (1.58–1.81n). Ploidy status was not correlated with *SDHx* mutations, tumor behavior (multifocality, metastasis, recurrence) or hereditary/sporadic tumor form. These findings align with prior reports showing no clear clinical correlation (20–23). Nevertheless, the small sample size of patients with poor prognosis in our series limits the power to reliably evaluate potential correlation between ploidy and tumor behavior.

In HNPGLs, the most prevalent type of structural chromosomal abnormality was deletion. Hemizygous deletions in the 1p36 locus were frequently observed, which is a mutational hotspot in various human malignancies, including pheochromocytoma, leiomyoma, neuroblastoma, ovarian cancer, and others (24–26). This region is of particular significance for PPGLs due to the presence of the *SDHB* tumor suppressor gene, which is located at 1p36.13. Deletions at the *SDHB* locus were found in 44% of HNPGLs, encompassing both hereditary and sporadic forms. Furthermore, all tumors exhibiting a germline *SDHB* mutation demonstrated somatic *SDHB* deletion, thus indicating two-hit inactivation. Other candidate tumor suppressors in the 1p36 region, including *CHD5*, *KIF1B*, and *CASZ1*, have been previously suggested (27–31). Our results additionally implicate potential tumor suppressor genes *SPEN*, *CASP9*, *PRDM2*, *ARHGEF10L*, *ARID1A*, and *ID3* identified as frequently deleted at the 1p36 region in HNPGLs.

Homozygous deletions, which are relatively rare events in cancer, typically result in biallelic inactivation of tumor suppressor genes (32). In the present study, we identified homozygous deletions affecting six tumor suppressor genes: *ATP2B3, BCL10, PHF6, RPL10, RPL5*, and *STAG2*. Although none of these genes has been previously implicated in PPGLs or other neuroendocrine neoplasms, their biallelic loss suggests a previously unrecognized tumor suppressor function in HNPGL tumorigenesis. Among these, *RPL5*, which encodes a component of 60S large ribosomal subunits (uL18), is of particular interest. Heterozygous inactivating deletions or mutations in *RPL5* have been recurrently documented in glioblastoma, melanoma, breast cancer, and multiple myeloma (33, 34). Furthermore, reduced *RPL5* mRNA expression has been associated with poorer overall survival in these cancers, and has been linked to responses to anticancer therapy in multiple myeloma (34). Deleterious missense mutations in the *RPL5* and *RPL10* genes have been reported in approximately 10% of pediatric T-cell acute lymphoblastic leukemia cases (35). Notably, in our study cohort, we also detected a homozygous deletion in *RPL10*, which encodes another 60S ribosomal subunit protein (uL16). Overall, homozygous deletions involving *RPL5* and/or *RPL10* were observed in 16% (4/25) of HNPGLs; including hemizygous deletions, alterations in these genes were found in 56% (14/25) of cases. This recurrent alteration of two large-subunit ribosomal protein genes suggests that disruption of ribosomal biogenesis may represent a pathogenic mechanism in HNPGL tumorigenesis, warranting further investigation into its functional consequences and potential therapeutic implications.

*STAG1* and *STAG2* encode components of the cohesin complex, which is essential for maintaining sister chromatid cohesion and chromosome segregation during cell division, as well as for genome organization, DNA repair, and gene expression regulation (36). In our study, we observed hemizygous or homozygous deletions of these genes in 40% (10/25) of HNPGLs, with *STAG2* deletions being the most frequent, accounting for 32% (8/25) of cases. *STAG2* is located on the X chromosome; consequently, the gene inactivation can be achieved through disruption of a single allele. Somatic inactivating mutations in *STAG2* have been identified in various human malignancies and are recognized as driver events in tumorigenesis (36, 37). *STAG2* inactivation has been shown to promote aneuploidy in both normal and malignant cells (38, 39). Likewise, *STAG1*, the paralog of *STAG2*, has been implicated in tumor aneuploidy and shown to be indispensable for cell survival in the absence of *STAG2* (40–42). Therefore, the recurrent deletions of *STAG1* and *STAG2* observed in our cohort may indicate a critical role for these genes in driving chromosomal instability in HNPGLs. Furthermore, pharmacological inhibition of *STAG1* in tumors harboring *STAG2* deletions could represent a promising selective therapeutic strategy for patients with HNPGLs.

Notably, other tumor suppressor genes mentioned above were also affected by both hemizygous and homozygous deletions, demonstrating a high mutation frequency in HNPGLs: *ATP2B3* (16%, 2/25), *BCL10* (44%, 11/25), and *PHF6* (32%, 8/25). These findings highlight candidate genes for future functional studies to clarify their involvement in HNPGL pathogenesis.

Hemizygous deletions frequently affected known PPGLs-related genes (*SDHB, SDHD, ATRX, SDHA, SDHAF2, FH*, and *VHL*) across both hereditary and sporadic tumors in this study. It has been widely accepted that germline mutations in PPGL disease-causing genes (e.g., *SDHx*) are mutually exclusive in the majority of cases (43). However, we frequently observed a germline variant in one *SDHx* gene and a somatic deletion in another. The co-occurrence of germline and somatic mutations in different genes has been previously reported in PPGLs (e.g., *SDHB*/*ATRX*, *SDHB*/*TERT*, and *SDHB*/*NF1*) (44–46). Such co-occurring alterations may increase tumor initiation risk, promote progression and metastasis through convergent or synergistic pathway effects, contribute to variable tumor phenotypes, and partially explain the incomplete penetrance observed in hereditary PPGLs.

Amplifications of genomic regions were observed less frequently than deletions. The most frequently identified amplifications, at 9q33.3-9q34.11, 7q36, and 1q41 - 1q42.11 loci, are not canonical for PPGLs or neuroendocrine tumors. However, amplifications within the 1q41 - 1q42.11 locus may hold clinical relevance, as 1q gain has been associated with aggressiveness and progression in multiple tumor types, including multiple myeloma, breast, esophageal, and hepatocellular carcinomas (47–50). In a subset of HNPGLs studied, we detected amplifications in well-characterized oncogenes (*BRAF*, *IDH1*, *KIT*, and *PDGFRA*) that are also involved in molecular pathways underlying PPGLs pathogenesis, including the pseudohypoxia pathway (*IDH1*) and the kinase signaling pathway (*BRAF*) (51). Activation of *KIT* and *PDGFRA* is a known driver of gastrointestinal stromal tumors (GISTs), which may rarely co-occur with PPGLs in certain hereditary syndromes (52). Importantly, all four genes have been explored as therapeutic targets in other malignancies, and their detection in our cohort may point to potential therapeutic strategies for specific HNPGL cases.

An additional notable finding concerned a set of tumor suppressor genes (*CARS*, *CBLB*, *PRDM2*, *SMARCA4*) and oncogenes (*CDKN1A*, *HGF*) harboring concurrent genetic alterations, mutations accompanied by deletions or amplifications, respectively. These genes predominantly carried germline missense variants classified as of uncertain significance according to the ACMG/AMP 2015 criteria (53). Mutations in *CARS* and *PRDM2* showed variant allele frequencies (VAFs) > 0.7, suggesting wild−type allele loss and potential biallelic inactivation. In contrast, the VAFs observed for *CBLB*, *SMARCA4*, *CDKN1A*, and *HGF* did not provide conclusive evidence for dominance of the mutant allele. The *CARS* gene encodes cysteinyl-tRNA synthetase 1 and is located at 11p15.4, in proximity to 11p15.5, a region harboring a major cluster of imprinted genes. Lai et al. demonstrated recurrent somatic loss of the 11p15.5-11p15.4 regions in PPGLs, particularly in *SDHD*-mutated tumors (54). Based on a model proposed by Hensen et al., an unknown paternally imprinted tumor suppressor gene at 11p15.5 has been suggested to drive tumor development in *SDHD*-mutated PPGLs (55). In our study, a *CARS* germline mutation was identified in a patient (067tc) with a germline *SDHD* mutation, and both genes exhibited hemizygous deletions. The VAF values for these mutations were 0.79 and 0.65, respectively, suggesting potential loss of the wild−type maternal alleles for both genes. On one hand, these findings raise the possibility that *CARS* may represent a tumor suppressor gene at the 11p15 locus (alongside *SDHD*) contributing to tumor formation through classical biallelic inactivation rather than through imprinting. On the other hand, the *CARS* gene harbored a variant of uncertain significance, and no data on biallelic gene loss in association with cancer are available, suggesting that the observed two−hit alteration may represent a secondary event rather than a primary driver.

In contrast, *PRDM2* (also known as *RIZ*), located at chromosome 1p36.21 and encoding several subtypes of nuclear zinc finger epigenetic regulator protein, represents a classical two−hit tumor suppressor implicated in various diseases and malignancies (56). Notably, *PRDM2* has been found to carry recurrent somatic deletions and to be significantly downregulated in PPGLs (57). In our study, two−hit alterations in *PRDM2* were detected in a patient with an apparently sporadic tumor, suggesting a potential role for this event in tumor development. Nevertheless, in the absence of functional validation, the true pathogenicity of the identified variants in these genes remains indeterminate, limiting definitive conclusions regarding biallelic gene inactivation.

A limitation of this study is the small sample size of high-purity tumors. This selection was methodologically necessary, as allele-specific CNA analysis via ASCAT requires high tumor cellularity to avoid false-negative results and ensure accurate ploidy estimation. However, in routine clinical practice, HNPGL biopsies often present with lower tumor purity due to vascular or stromal contamination, which may restrict the direct application of this approach to all clinical specimens. Nevertheless, this is the largest HNPGL cohort to undergo allele-specific CNA analysis. Future directions include validating these findings in larger cohorts and conducting functional analyses in relevant cell lines (e.g., PC12). A technical limitation of this study is the unreliable CNA estimation within pericentromeric regions, including 9p11.2–9q21.11, 21p11.1–21q11.2, and 7q11. These loci consist of highly repetitive sequence elements that cause ambiguous read alignment during standard NGS analysis. To mitigate this, we applied the mappability-aware preprocessing function (ascat.prepareHTS) within the ASCAT algorithm. However, this correction only partially reduces the noise and cannot fully resolve the inherent ambiguity in these regions. Consequently, any CNAs identified at these specific coordinates should be interpreted with caution and require orthogonal validation using an independent technique not affected by mappability biases, such as FISH or digital PCR.

## Conclusion

The present study reveals a complex and heterogeneous CNA landscape in HNPGLs, underscoring a previously underappreciated level of genetic instability in these tumors. The CNA profiles are characterized by a high prevalence of deletions and recurrent alterations affecting a wide spectrum of PPGL susceptibility and candidate driver genes, including *SDHB*, *SDHD*, *ATRX*, *SDHAF2*, *FH*, *MEN1*, *SDHA*, *VHL*, *BRAF*, *IDH1*, *PDGFRA*, and *KIT*. These findings expand the understanding of HNPGL molecular pathogenesis and implicate a broader set of genes in tumor development than previously recognized. Several identified alterations may have potential clinical value for prognosis (e.g., 1q gain) and personalized therapy (e.g., *STAG1*/*STAG2* deletion, *PDGFRA* and *KIT* amplifications).

## Data Availability

The raw data supporting the conclusions of this article will be made available by the authors, without undue reservation.
