# Supplementary material for: Complex landscape of somatic copy number alterations in head and neck paragangliomas
Source: Front Endocrinol (Lausanne). 2026 Aug 14;17:1841009. doi: 10.3389/fendo.2026.1841009 (PMC13521833; doi:10.3389/fendo.2026.1841009)
Supplement: Supplementary file 1 [file DataSheet1.pdf]

tumor id = 001tc1

Ploidy: 2.01, purity: 100%, goodness of fit: 91.6%, non-aberrant

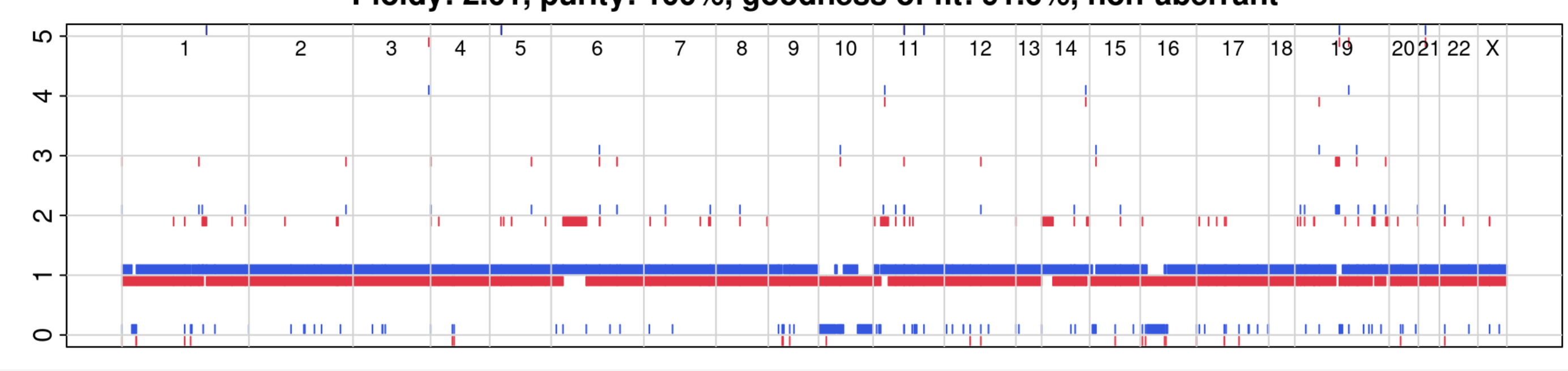

tumor id = 001tc2

Ploidy: 1.99, purity: 83%, goodness of fit: 86.6%, non-aberrant

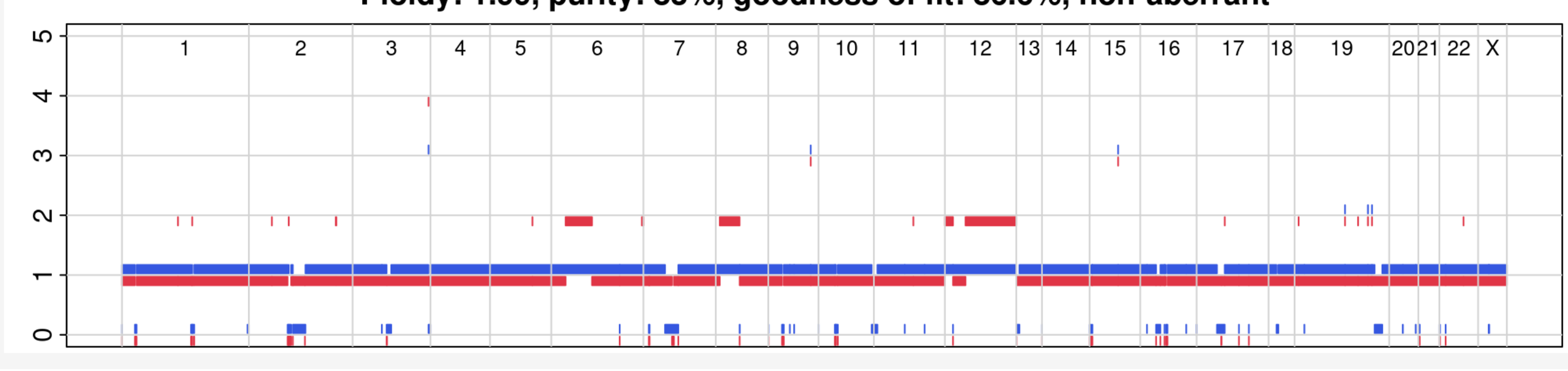

tumor id = 001tv

Ploidy: 2.18, purity: 76%, goodness of fit: 89.9%, non-aberrant

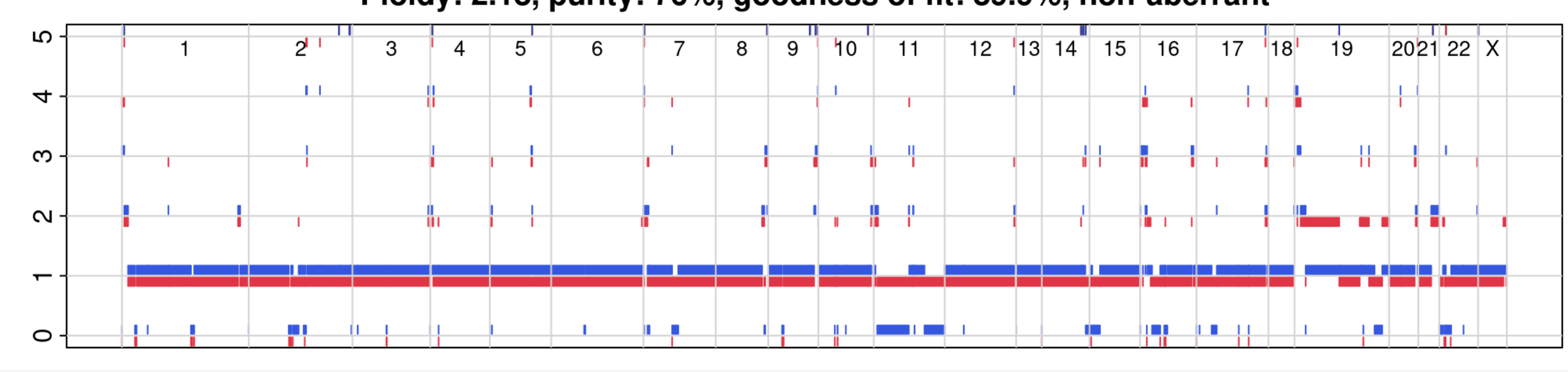

tumor id = 024tc

Ploidy: 1.98, purity: 90%, goodness of fit: 88.9%

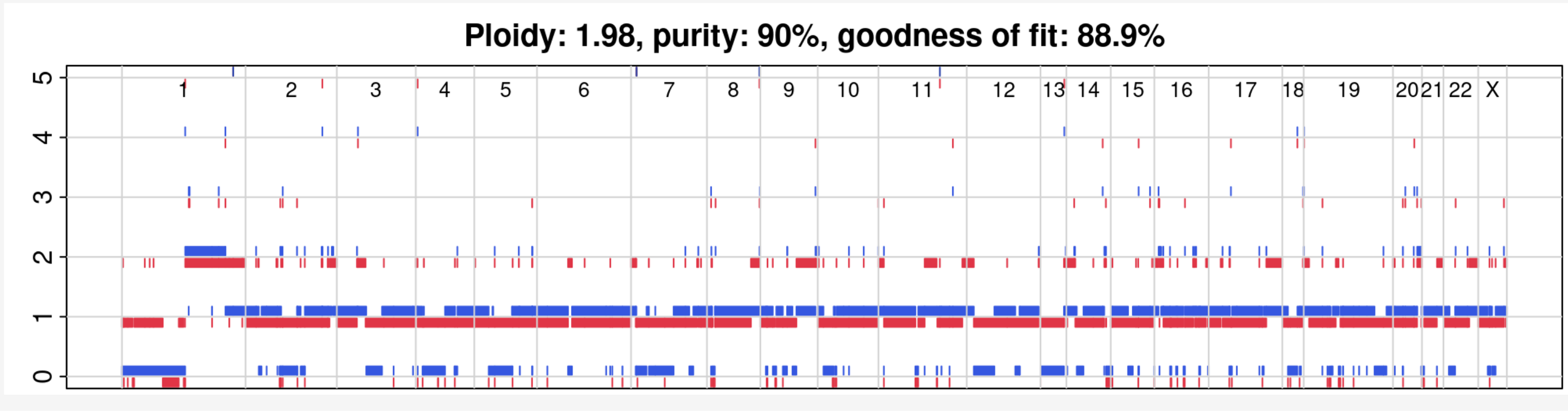

tumor id = 026tv

Ploidy: 1.97, purity: 100%, goodness of fit: 88.7%, non-aberrant

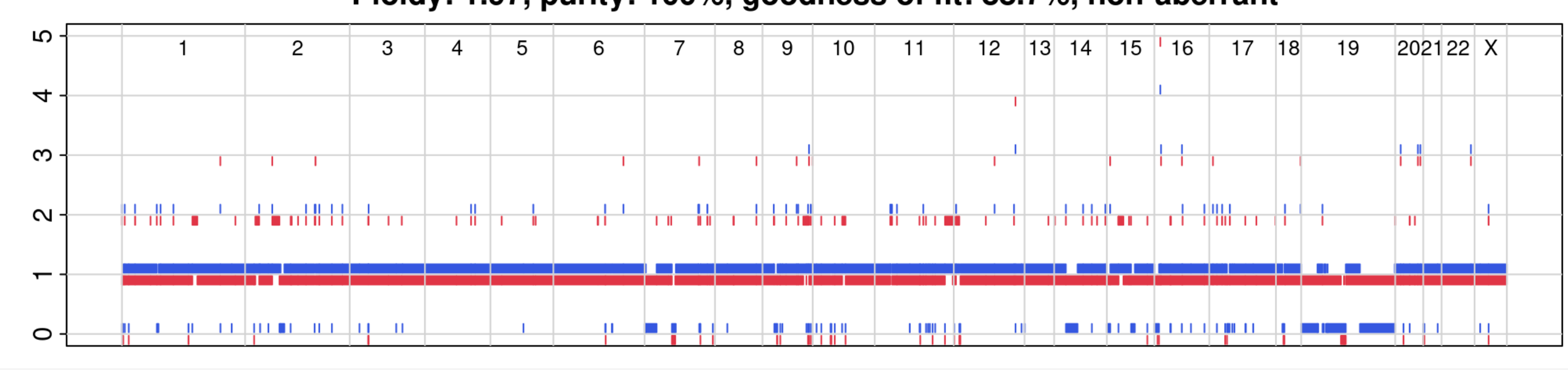

tumor id = 062tc

Ploidy: 1.97, purity: 83%, goodness of fit: 87.9%, non-aberrant

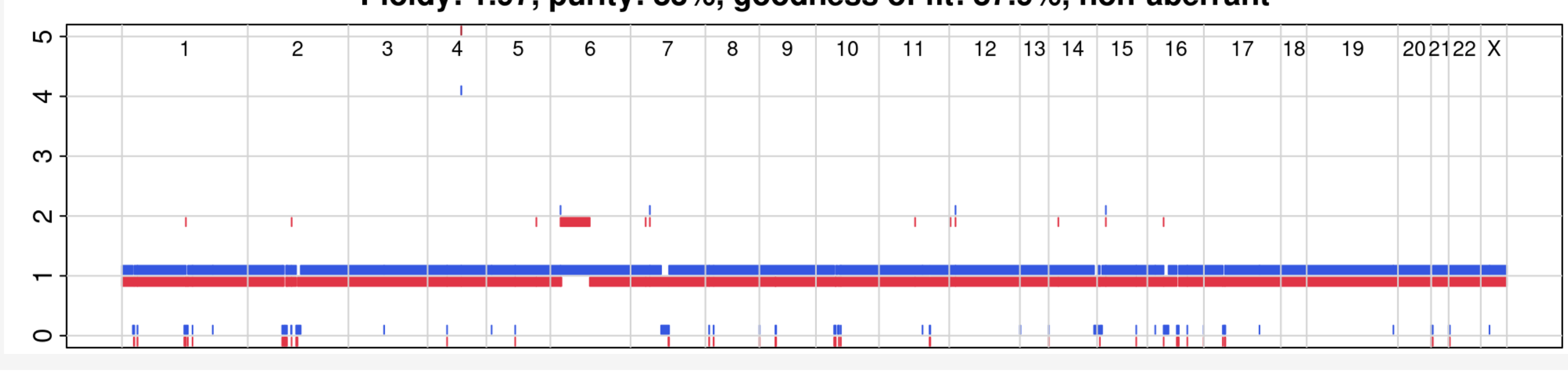

tumor id = 065tc

Ploidy: 4.29, purity: 100%, goodness of fit: 94.5%

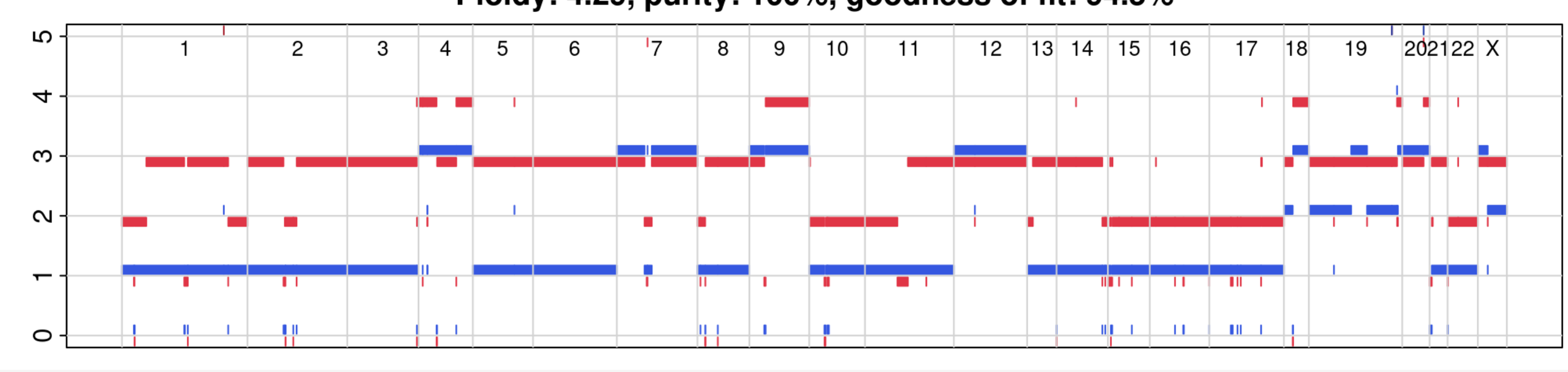

tumor id = 066tc

Ploidy: 4.54, purity: 71%, goodness of fit: 86.4%

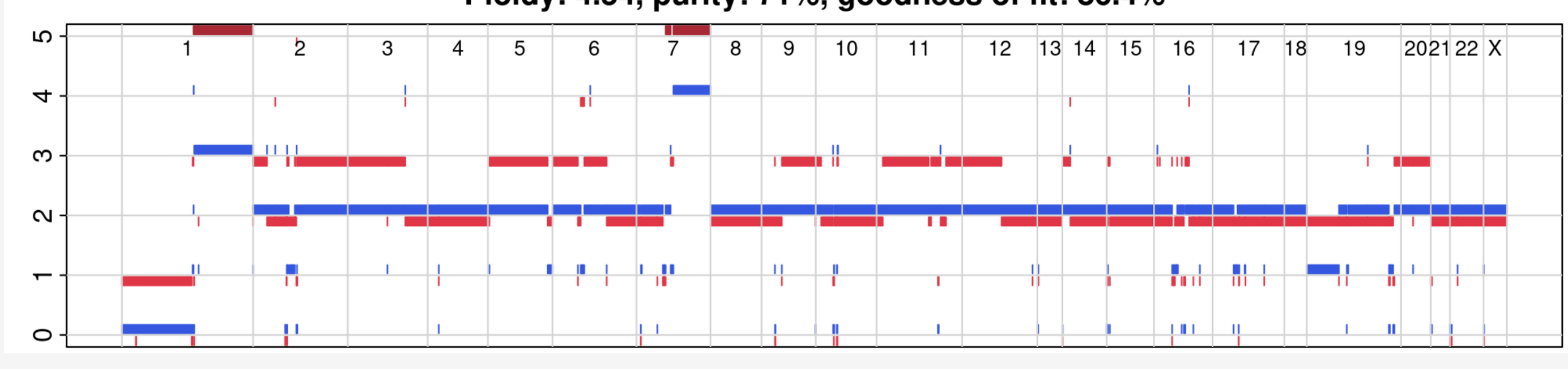

tumor id = 067tc

Ploidy: 1.81, purity: 79%, goodness of fit: 84.4%, non-aberrant

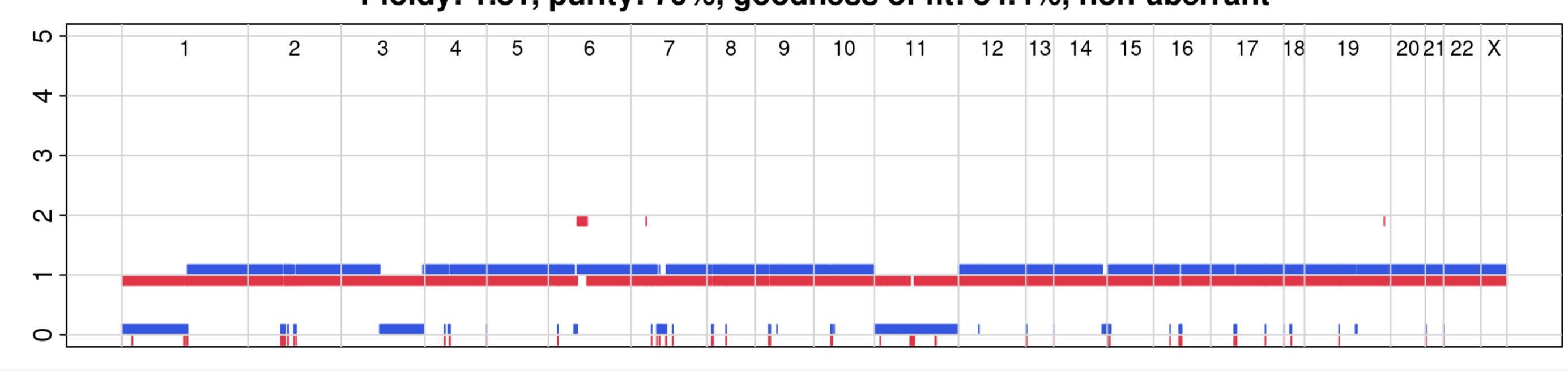

tumor id = 072tc

Ploidy: 1.80, purity: 89%, goodness of fit: 90.2%

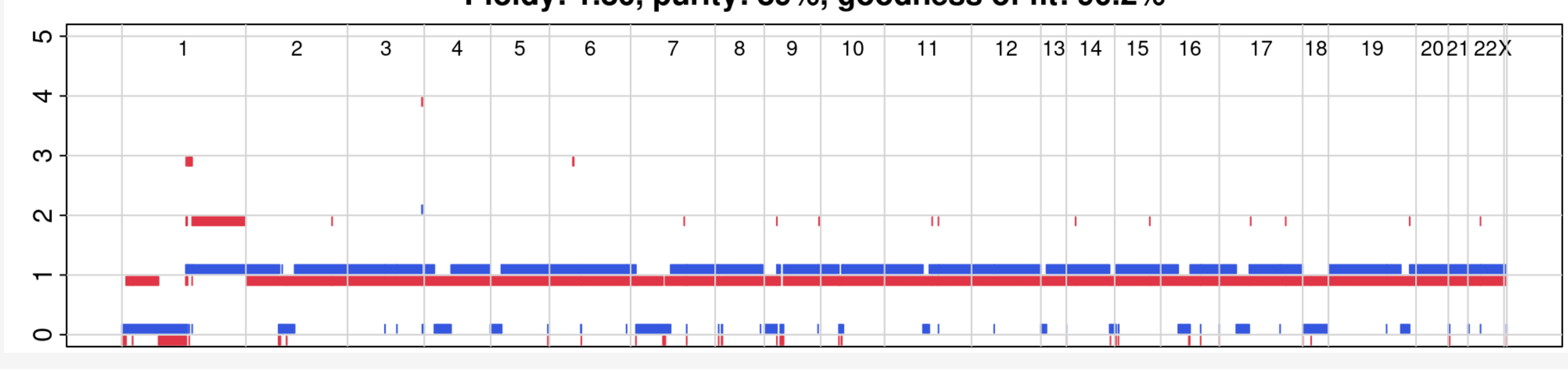

tumor id = 082tc1

Ploidy: 2.21, purity: 99%, goodness of fit: 91.5%, non-aberrant

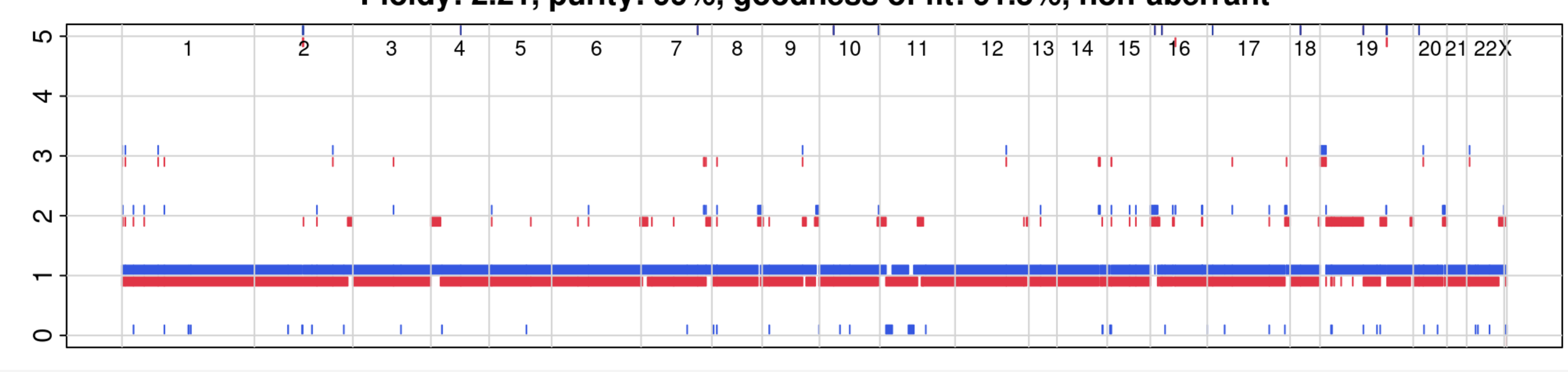

tumor id = 082tv

Ploidy: 2.06, purity: 99%, goodness of fit: 93.9%, non-aberrant

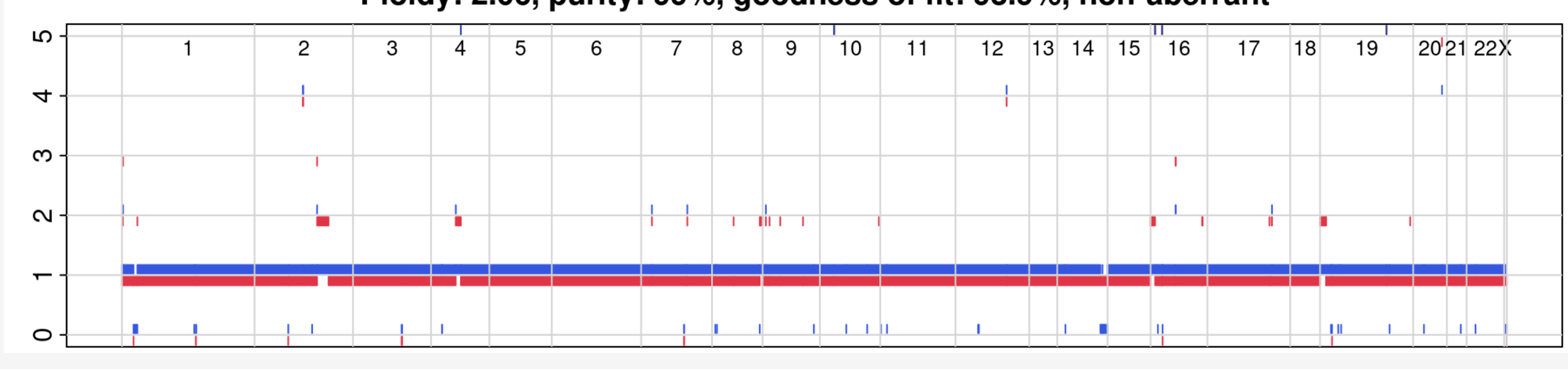

tumor id = 100tc

Ploidy: 1.73, purity: 76%, goodness of fit: 81.9%

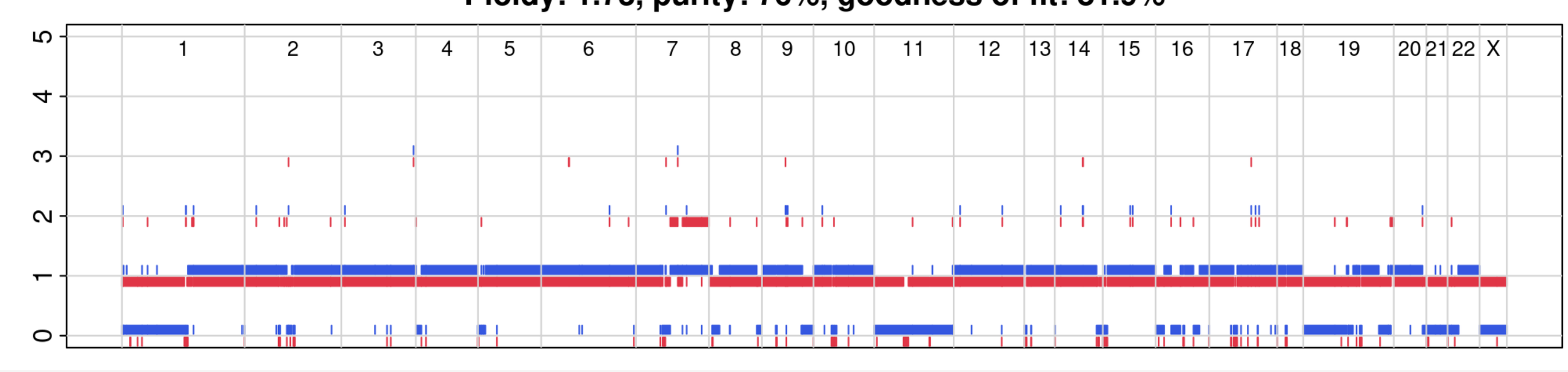

tumor id = 101tv1

Ploidy: 1.93, purity: 78%, goodness of fit: 89.5%, non-aberrant

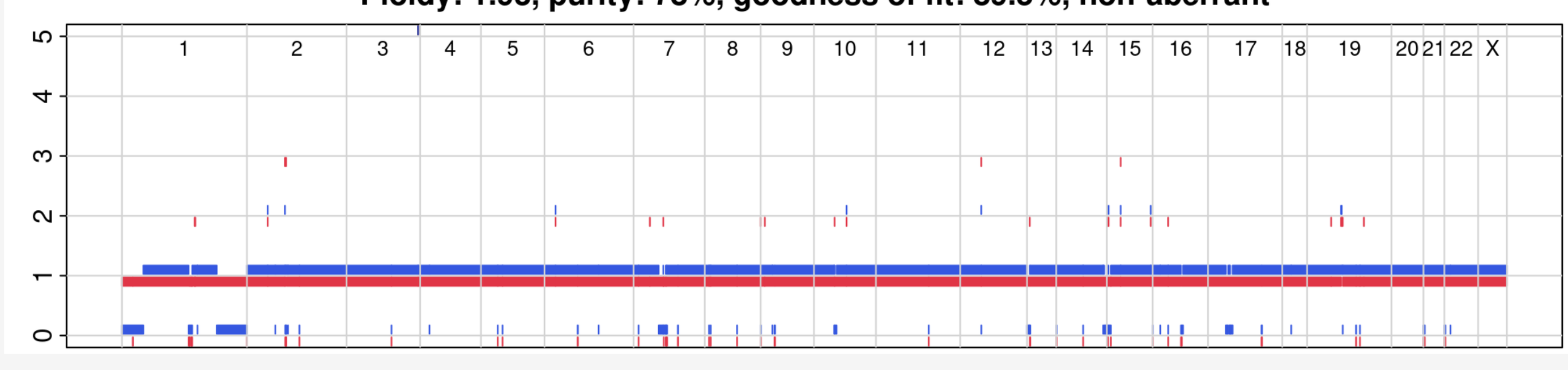

tumor id = 101tv2

Ploidy: 1.95, purity: 81%, goodness of fit: 80.4%

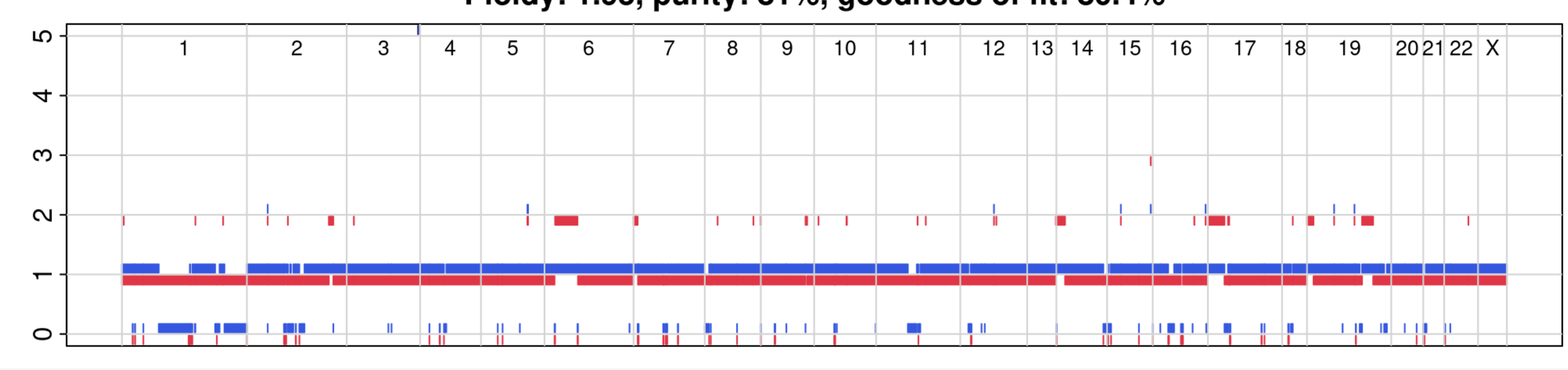

tumor id = 103tc

Ploidy: 1.93, purity: 90%, goodness of fit: 91.8%, non-aberrant

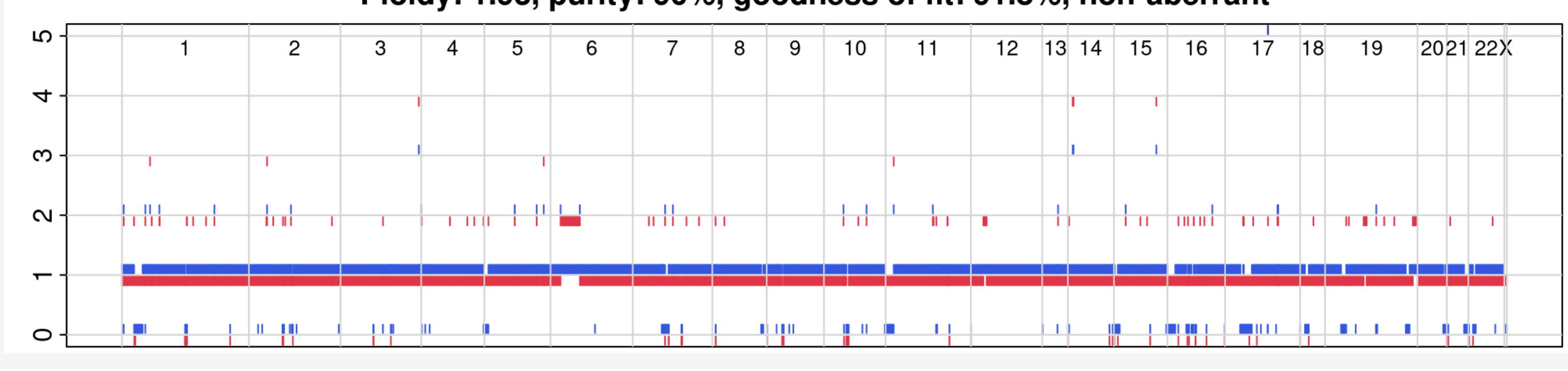

tumor id = 105tc

Ploidy: 1.77, purity: 81%, goodness of fit: 89.7%

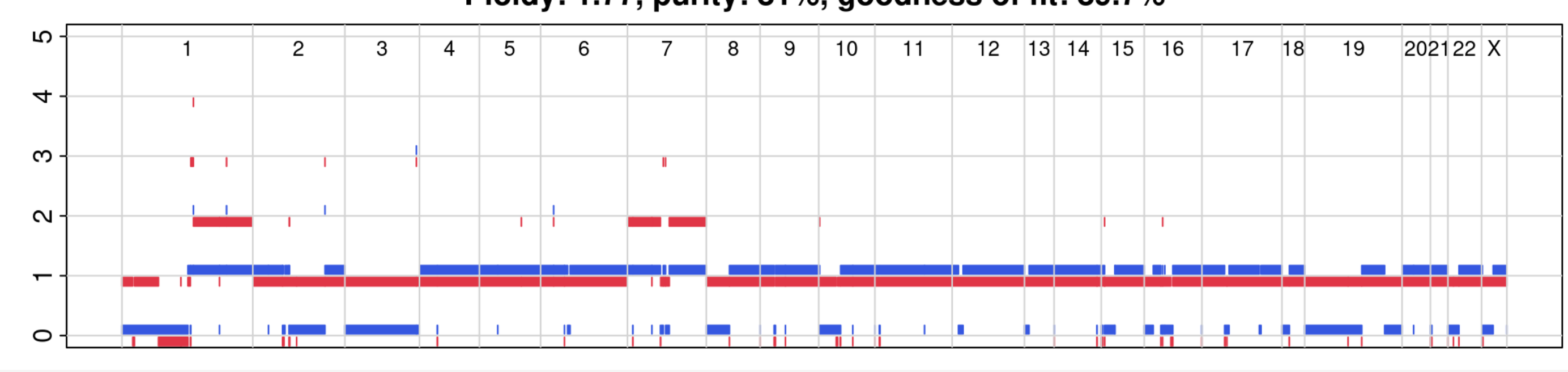

tumor id = 106tc

Ploidy: 1.97, purity: 81%, goodness of fit: 99.3%

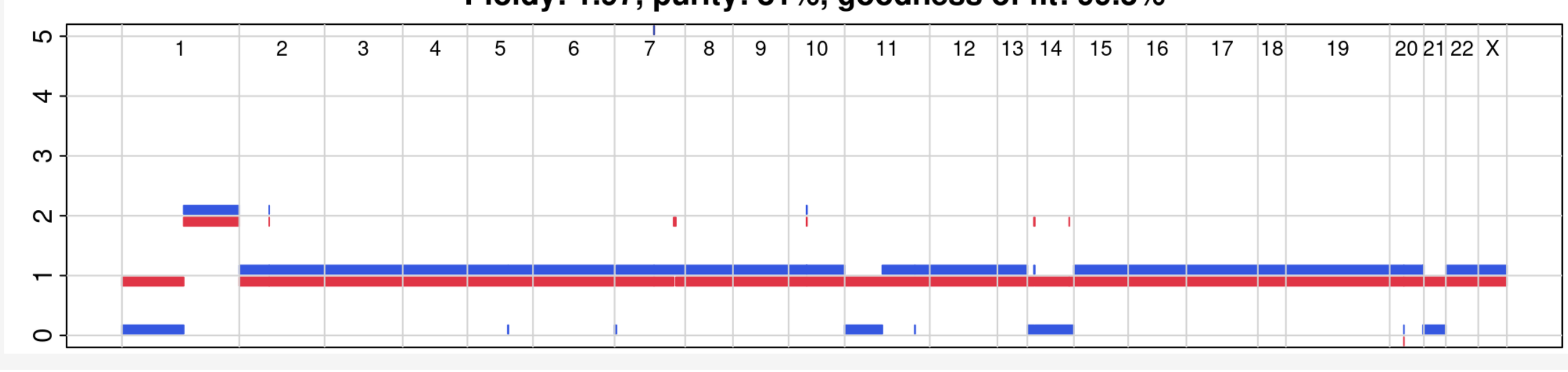

tumor id = 118tv

Ploidy: 2.06, purity: 99%, goodness of fit: 96.9%, non-aberrant

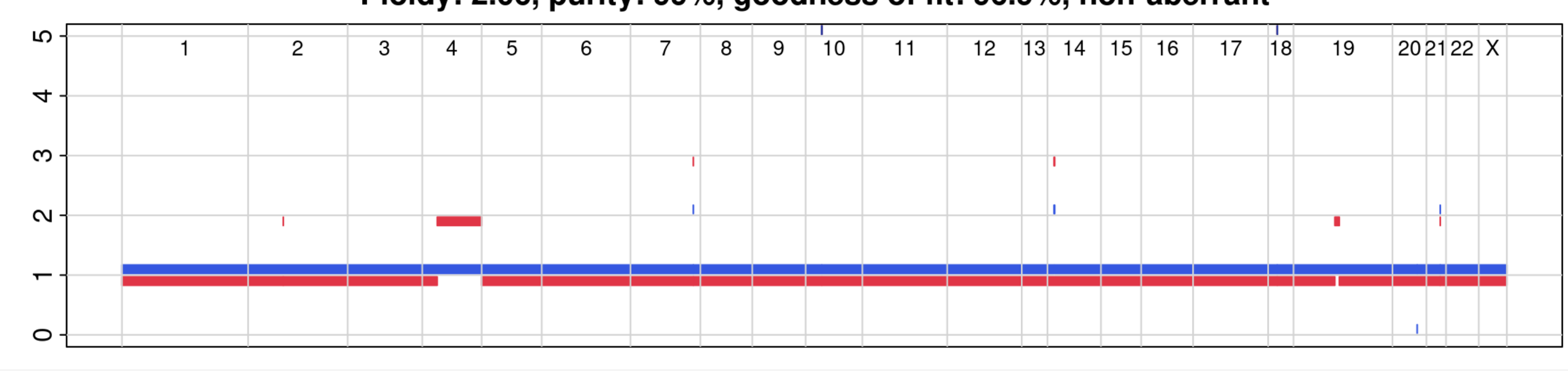

tumor id = 120tv

Ploidy: 1.58, purity: 73%, goodness of fit: 90.8%

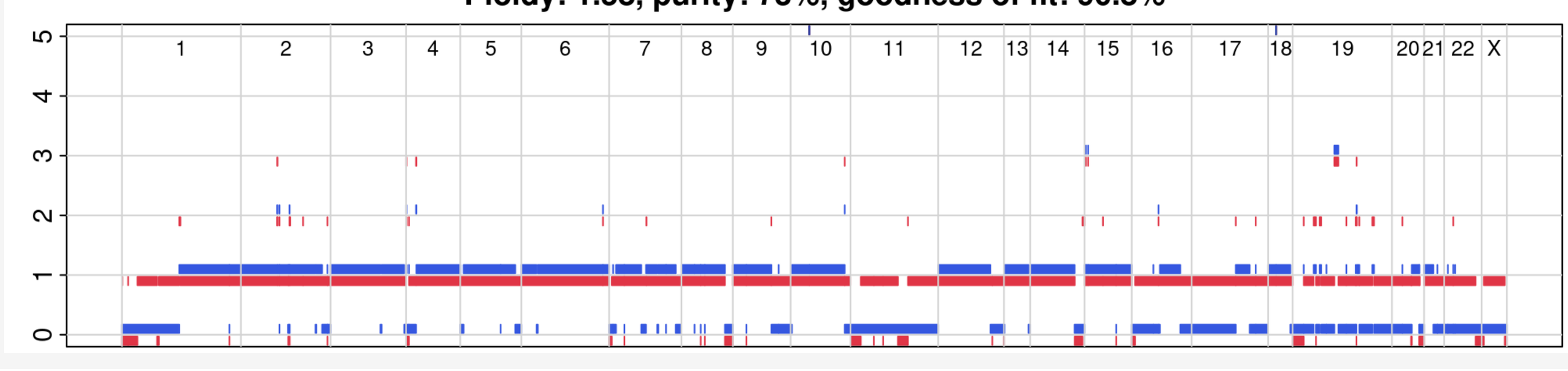

tumor id = 123tv

Ploidy: 1.61, purity: 82%, goodness of fit: 82.1%

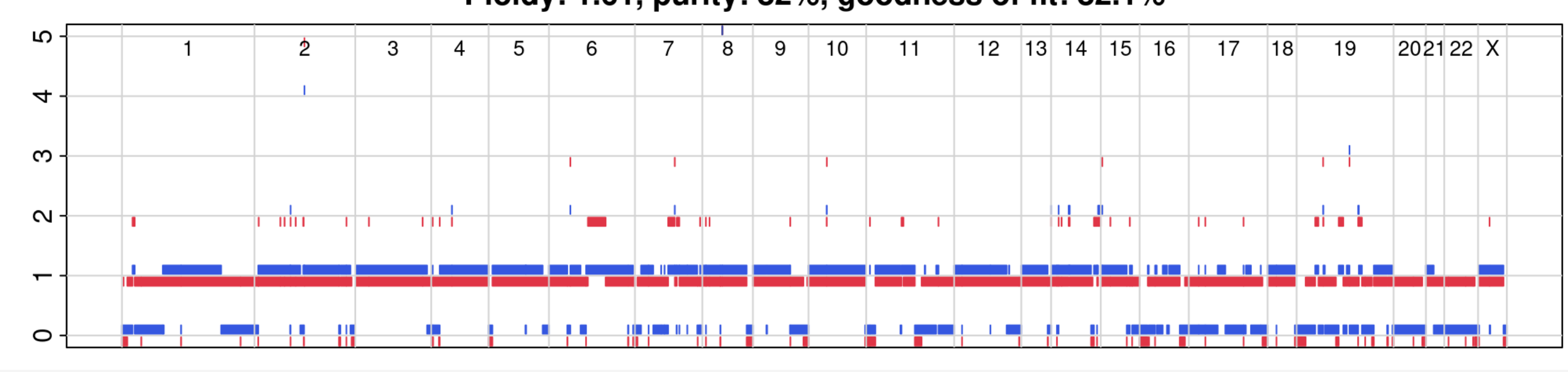

tumor id = 125tv

Ploidy: 2.67, purity: 73%, goodness of fit: 98.9%

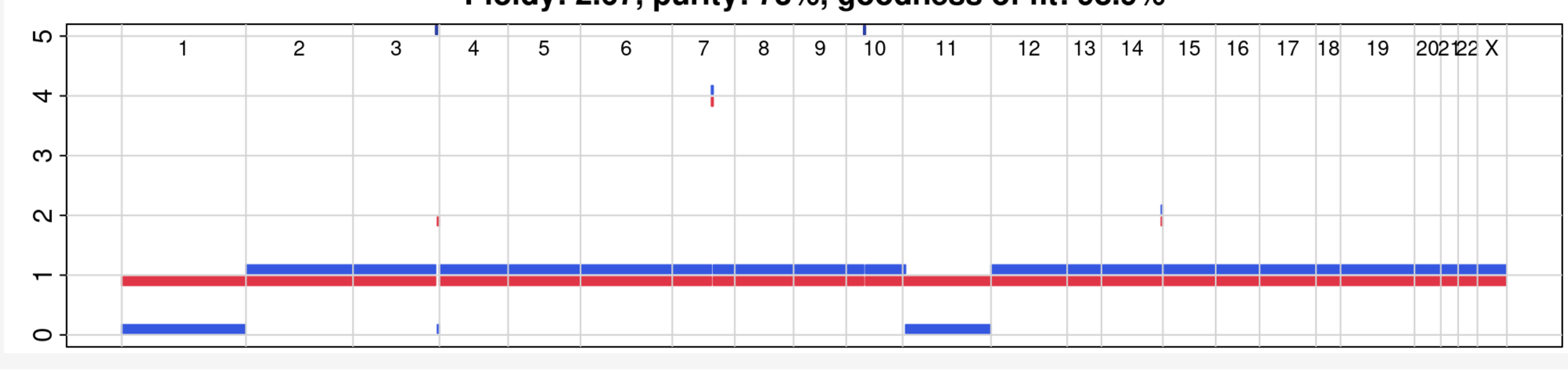

tumor id = 143tc

Ploidy: 2.15, purity: 100%, goodness of fit: 88.1%, non-aberrant

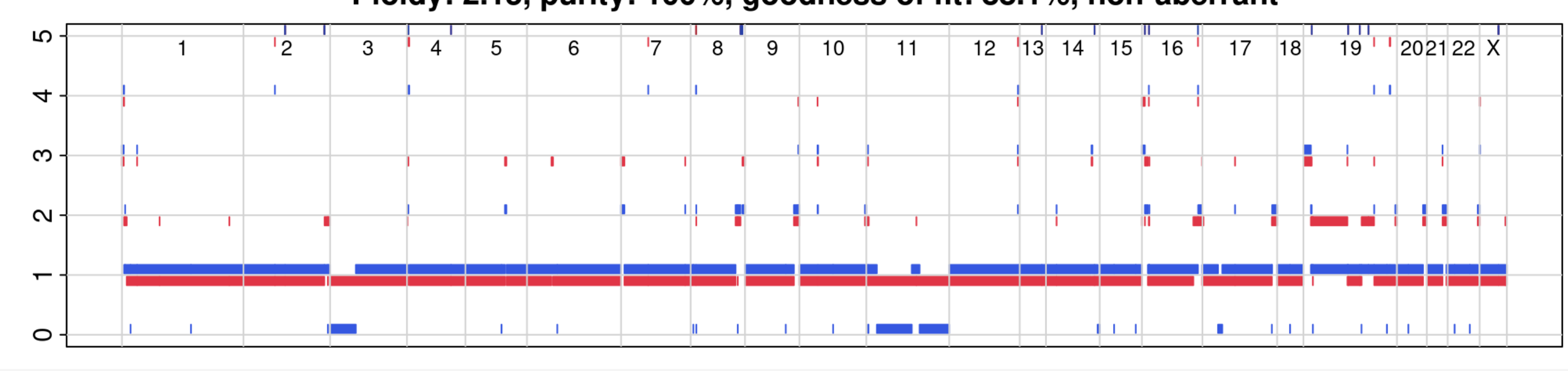

tumor id = 149tc

Ploidy: 2.01, purity: 100%, goodness of fit: 97.3%, non-aberrant

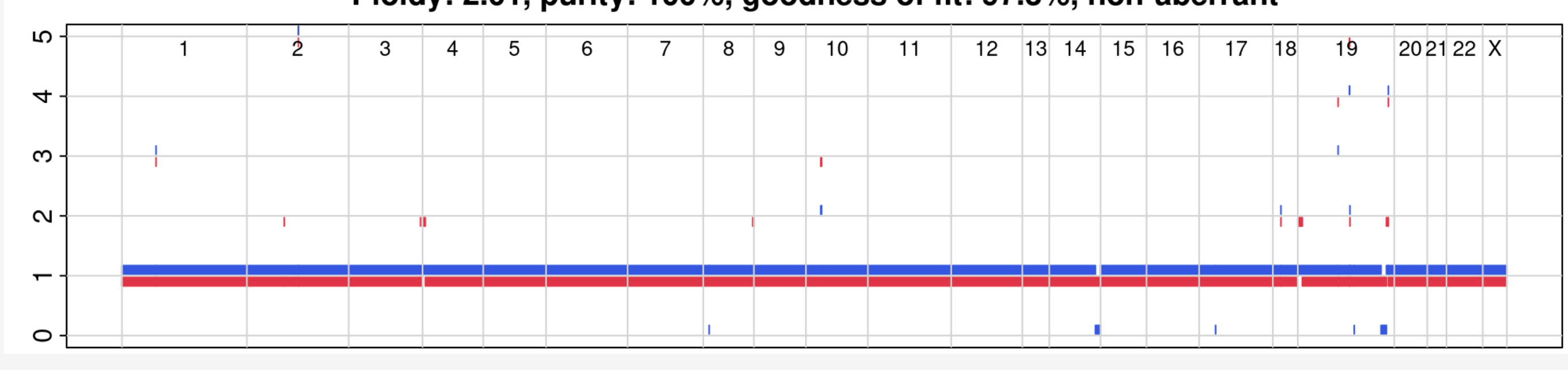

tumor id = 157tc

Ploidy: 2.28, purity: 100%, goodness of fit: 87.1%, non-aberrant

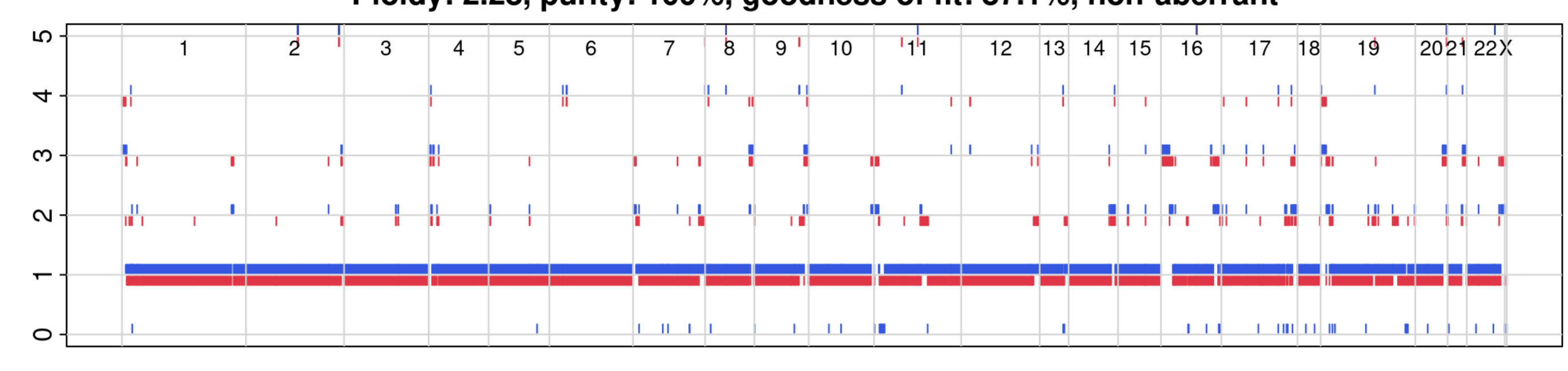

Supplementary File 1. Cohort−wide Allele−Specific Copy Number Profiles. Copy number is plotted on the y−axis against the genomic location on the x−axis, with the allele of the lowest copy number shown in blue and the highest in red.
