# Supplementary material for: Complex landscape of somatic copy number alterations in head and neck paragangliomas
Source: Front Endocrinol (Lausanne). 2026 Aug 14;17:1841009. doi: 10.3389/fendo.2026.1841009 (PMC13521833; doi:10.3389/fendo.2026.1841009)
Supplement: Supplementary file 2 [file DataSheet3.docx]

**Figure S1.** Distribution and frequency of tumor suppressor gene deletions in HNPGLs. The strip plot displays tumor suppressor genes affected by hemizygous (blue) and homozygous (black) deletions in at least 28% of samples (n ≥ 7). Genes exhibiting dual functionality (oncogene and tumor suppressor) are denoted by blue letters. Gene classification is based on the Cancer Gene Census (COSMIC).

**Figure S2.** Distribution and frequency of oncogene amplifications in HNPGLs. The strip plot displays oncogenes with asymmetric (red) or local balanced (purple) amplifications in at least 12% of samples (n ≥ 3). Genes exhibiting dual functionality (oncogene and tumor suppressor) are denoted by blue letters. Gene classification is based on the Cancer Gene Census (COSMIC).

**Figure S3.** The loss of heterozygosity (gray) near the *SDHB* and *SDHD* gene loci in hereditary HNPGLs. MUT, mutation; Hom, homozygous non-informative microsatellite.

**Table S1.** Somatic copy number alterations associated with apparently sporadic HNPGLs.

| **Chr** | **Start** | **Stop** | **Length (nt)** | **Cytoband** | **Event** | **Number of tumors, % (n)** | ***Р*-value** | **COSMIC gene** |
| --- | --- | --- | --- | --- | --- | --- | --- | --- |
| 1 | 216173861 | 224345109 | 8171249 | 1q41-1q42.11 | Asymmetric allele amplifications | 50% (4/8) | 0.0055 | - |
| 1 | 228560181 | 246939921 | 18379741 | 1q42.13-1q44 | Asymmetric allele amplifications | 50% (4/8) | 0.0055 | *AKT3*, *FH*, *RGS7* |
| 1 | 196876624 | 204379617 | 7502994 | 1q31.3-1q32.1 | Asymmetric allele amplifications | 50% (4/8) | 0.0055 | *ASPM*, *BTG2*, *ELF3*, *PTPRC* |
| 1 | 55539557 | 104117762 | 48578206 | 1p32.3-1p21.1 | Homozygous deletions | 37.5% (3/8) | 0.02 | *BCL10*, *FUBP1*, *JAK1*, *JUN*, *RPL5* |
| 3 | 48289176 | 56592926 | 8303751 | 3p21.31-3p14.3 | Asymmetric allele amplifications | 37.5% (3/8) | 0.02 | *BAP1*, *CACNA1D*, *NCKIPSD*, *PBRM1*, *RHOA* |
| 11 | 124524793 | 134856626 | 10331834 | 11q24.2-1q25 | Asymmetric allele amplifications | 37.5% (3/8) | 0.02 | *FLI1*, *KCNJ5* |
| 11 | 118430519 | 129804950 | 11374432 | 11q23.3-11q24.3 | Asymmetric allele amplifications | 37.5% (3/8) | 0.02 | *ARHGEF12*, *BCL9L*, *CBL*, *DDX6*, *FLI1*, *FOXR1*, *KCNJ5* |
